# PeakRegressor Identifies Composite Sequence Motifs Responsible for STAT1 Binding Sites and Their Potential rSNPs

**DOI:** 10.1371/journal.pone.0011881

**Published:** 2010-08-27

**Authors:** Jean-François Pessiot, Hirokazu Chiba, Hiroto Hyakkoku, Takeaki Taniguchi, Wataru Fujibuchi

**Affiliations:** 1 Computational Biology Research Center, Advanced Industrial Science and Technology (AIST), Tokyo, Japan; 2 Waseda University, Tokyo, Japan; 3 Mitsubishi Research Institute, Inc., Tokyo, Japan; National Cancer Institute at Frederick, United States of America

## Abstract

How to identify true transcription factor binding sites on the basis of sequence motif information (e.g., motif pattern, location, combination, etc.) is an important question in bioinformatics. We present “PeakRegressor,” a system that identifies binding motifs by combining DNA-sequence data and ChIP-Seq data. PeakRegressor uses L1-norm log linear regression in order to predict peak values from binding motif candidates. Our approach successfully predicts the peak values of STAT1 and RNA Polymerase II with correlation coefficients as high as 0.65 and 0.66, respectively. Using PeakRegressor, we could identify composite motifs for STAT1, as well as potential regulatory SNPs (rSNPs) involved in the regulation of transcription levels of neighboring genes. In addition, we show that among five regression methods, L1-norm log linear regression achieves the best performance with respect to binding motif identification, biological interpretability and computational efficiency.

## Introduction

The experimental identification of *cis*-regulatory sites based on transcription factor binding motifs (TFBMs) is a difficult and time-consuming task. In this regard, *in silico* analysis of TFBMs has recently attracted attention as a promising tool for discovering true *cis*-regulatory sites. Previous works attempt to find TFBMs to model the mechanisms underlying the control of gene expression levels [1], [2]. They assume that the gene expression levels are determined by the presence of certain motifs in the upstream regions of the genes. Based on this assumption, they find TFBM candidates which show a strong correlation with changes in the gene expression levels. [3] Instead of modeling the expression levels, another solution is to model the binding affinities between a protein and its target genes based on the thermodynamics theory. However, the binding affinities are difficult to measure and related works use transcription factor occupancy to approximate binding affinity [4], [5].

In this article, we present PeakRegressor, a new tool for the identification of functional TFBMs from ChIP-Seq data. As far as we know, this is the first attempt at performing peak signal regression based on candidate motif models. Because PeakRegressor is computationally efficient and the models are easy to interpret, it is usable with large-scale datasets. We apply PeakRegressor to two ChIP-Seq datasets and show its ability to recover motifs involved in the binding of STAT1 and RNA Polymerase II.

## Results and Discussion

### Results with PeakRegressor

Table 1 shows the correlation coefficients between the peak scores and their predicted values by PeakRegressor in the test dataset. We keep the highest correlation coefficient among various 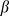 for each iteration of the 30-fold cross-validation, and those 30 correlation coefficients are averaged and shown here. Obviously, the filtering with peak existence probability, i.e., Q-value, over the control experiment enhances the regressions. The filtering with promoter region proximity improves the regressions of RNA Polymerase II but not of STAT1.

**Table 1:** Influence of the peak filtering methods on the correlation coefficients between peak values and their predicted values in the test dataset.

*Filtering method*	*#Peaks (STAT1/Pol II)*	*STAT1*	*Pol II*
None	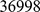 / 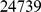	0.50	0.44
Promoter proximity	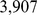 / 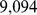	0.41	0.53
Q-value 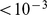	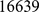 / 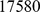	**0.65**	**0.66**

The correlation coefficients are averaged in 30-fold cross-validation.

**Figure 1:**
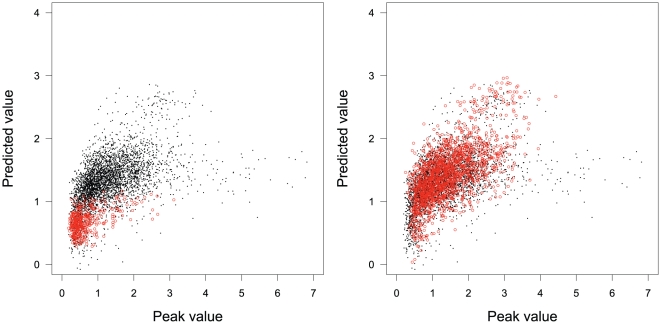
STAT1 regression results with two filtering methods: Q-value (right) and promoter proximity (left). The correlation coefficients on the test data between peak values and their predicted values are 0.65 and 0.41 for Q-value and promoter proximity filterings, respectively.

In Figure 1, we plot the STAT1 peak scores with two filtering methods such as Q-value 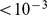 and promoter proximity in the test dataset against their predictions by PeakRegressor. The correlation coefficient is as high as 0.65 between the peak and predicted values for the Q-value filtering, whilst it is as low as 0.41 for promoter proximity filtering. Interestingly, however, the data points that are selected by promoter proximity existed only in a biased region, leading to worse prediction.

In Tables 2 and 3, we show the top ten motifs for STAT1 and RNA Polymerase II identified by PeakRegressor, respectively. The motifs are sorted according to the absolute values of their averaged regression coefficients. A motif with a positive (resp. negative) coefficient is thought to have a strengthening (resp. weakening) effect on the binding. In the case of STAT1, it is clear that our approach correctly identifies the classical GAS motif TTC[TC]N[GA]GAA as the main binding motif [6]. Meanwhile, the RNA Polymerase II binding motifs also contain known Downstream Promoter Element [AG]G[AT][CT][GAC] and Initiator Site [TC][TC]AN[TA][TC][TC] [7].

**Table 2:** List of putative STAT1 binding motifs identified by PeakRegressor.

*STAT1*	*Normalized coef.*
CA[TC]GTGACT[TG]C	1.
[TG]G[GTA][GC][AG]T**TT[CA]C[AGC][GA]GAA**[AC][TG]G[GA][GC]	0.96
**TTC[CT][TG][GA]GAA**AT[GC][CA][CA][CAT][AT][TCG][CG][CT]	0.72
[CT][TC]CA[GT]**TTCCAGGAA**[AT]T[CG][CAT]C[CT]	0.65
GGAGGGCG	−0.57
GGACGCCG	−0.56
A[CT]**TTC[TC][TG]GGAA**	0.56
**TT[CA]C[TAG][GA]GAA**[GA]T	0.55
A[TA]**TTCC[CT][GA]GAA**[AC]T[CG][AC]	0.48
**TT[CA][TC][GA]GGAA**[AG]	0.47

The classical GAS motifs are shown in boldface.

**Table 3:** List of putative RNA Polymerase II binding motifs identified by PeakRegressor.

*Pol II*	*Normalized coef.*
T[AG]**A[GC][TAG]CA**[GCT]A[AC]AA	1.
A[GA]AA[AC][CA]AA[AC]AAA	0.78
C[ACT]**[GT][CG][CT][TA]C**C[AGT]CC[TA]	0.76
C[CT][CG][AT]GGCTGG[AG]G	0.68
TTTCTGC[CT][CT]TT[GT]	0.67
T[TA]T[TC]**[CA]CAGACT**[AT]	0.63
GGAGGGAGGC[AG]G	0.62
AC[AC][CA][AC][AT][AG]AGAAA	0.61
TTTG**T[CT][TA]T[TG][AC][AT]**T	0.54
AAA[AT][GC]AAA[AT]A[GA]A	0.54

The known Downstream Promoter Element and Initiator site motifs are shown in boldface.

#### STAT1 composite motifs

As the most important feature of PeakRegressor, it can give us a list of putative composite motifs. Basically, it is difficult to evaluate whether a composite motif consists of the same motif or multiple (different) motifs. In order to identify the composite motifs, we proceed as follows. First, we consider the best set of motifs according to PeakRegressor (i.e., the set which corresponds to the best prediction accuracy). Among these, we select 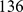 motifs which have a normalized coefficient higher than 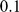. We use these motifs to represent each peak sequence as a binary vector, indicating whether a motif is present or not in the peak sequence. Then we cluster the resulting peak vectors using the K-Means algorithm. Thus each cluster contains peak vectors which show similar motif patterns, i.e., sequences containing potential composite motifs.

Here we show an example of a composite motif that are responsible for STAT1 binding signals:
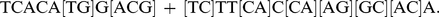



### Comparison with other regression methods

PeakRegressor identifies potential TFBMs by solving a regression problem. This regression problem is defined by a set of peak vectors 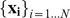 and their corresponding peak scores 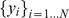. The goal is to predict the peak scores from the peak vectors. The fitted regression model is then used to infer the TFBM candidates. We expect the regression method to have three properties. First, it should identify the true binding motifs. Second, it should identify the strengthening and weakening motifs. Third, it should be computationally efficient in order to cope with large ChIP-Seq datasets.

In PeakRegressor, we choose to use the L1-norm log linear regression to solve this problem. This approach favors sparse solutions (i.e., solutions with a small number of motifs) and therefore, we argue that it is more suitable for the TFBM identification problem. However, many other regression methods are available and can be used to solve the regression problem. How do these approaches compare with the L1-norm log linear regression with respect to the desired properties? In the following, we compare our L1-norm log linear regression based approach with other regression methods: linear least squares regression, ridge regression, partial least squares regression, and principal component regression. For each method, we evaluate its performance on the STAT1 and RNA Polymerase II datasets and discuss the results.

#### Linear least squares regression

In Tables 4 and 5, we show the top ten motifs identified by the linear least squares regression. In the case of STAT1 (Table 4), we can see that the true GAS motif appears within the top ten motifs. However, two problems appear. First, the regression coefficients of the GAS motif are very low compared to those of the top motifs (between 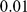 and 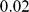). This means that according to the linear least squares regression, the true GAS motif has only a minor effect on the binding, which contradicts existing biological knowledge. Second, the most important motifs according to the linear least squares regression are CCCCTCCC and CCCCACCC. However, each of them is associated with opposite coefficients (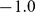 and 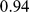 for CCCCTCCC, 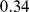 and 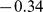 for CCCCACCC). Therefore, each of them is considered to have both a strengthening effect and a weakening effect on the binding, which is a contradictory result.

**Table 4:** List of putative STAT1 binding motifs identified by linear least squares regression.

*STAT1*	*Normalized coef.*
CCCCTCCC	−1.0
CCCCTCCC	0.94
CCCCACCC	0.34
CCCCACCC	−0.34
CA[TC]GTGACT[TG]C	0.02
[TG]G[GTA][GC][AG]T**TT[CA]C[AGC][GA]GAA**[AC][TG]G[GA][GC]	0.02
[CT][TC]CA[GT]**TTCCAGGAA**[AT]T[CG][CAT]C[CT]	0.01
GGAGGGCG	−0.01
**TTC[CT][TG][GA]GAA**AT[GC][CA][CA][CAT][AT][TCG][CG][CT]	0.01
A[CT]**TTC[TC][TG]GGAA**	0.01

The classical GAS motifs are shown in boldface.

**Table 5:** List of putative RNA Polymerase II binding motifs identified by linear least squares regression.

*RNA Polymerase II*	*Normalized coef.*
T[AG]**A[GC][TAG]CA**[GCT]A[AC]AA	1.0
A[GA]AA[AC][CA]AA[AC]AAA	0.86
C[ACT]**[GT][CG][CT][TA]C**C[AGT]CC[TA]	0.81
C[CT][CG][AT]GGCTGG[AG]G	0.74
TTTCTGC[CT][CT]TT[GT]	0.74
GGAGGGAGGC[AG]G	0.69
AC[AC][CA][AC][AT][AG]AGAAA	0.64
T[TA]T[TC]**[CA]CAGACT**[AT]	0.62
TTTG**T[CT][TA]T[TG][AC][AT]**T	0.62
**TT[TAC]TTT[CT]**TT[TC]TT	−0.61

The known Downstream Promoter Element and Initiator site motifs are shown in boldface.

With the RNA Polymerase II dataset (Table 5), linear least squares regression is able to identify the initiator site and the downstream promoter element. However, the instances of the initiator site have opposite coefficients ([CA]CAGACT with 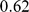, T[CT][TA]T[TG][AC][AT] with 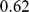, and TT[TAC]TTT[CT] with 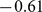). As they are instances of the same motif, we expect them to have the same sign i.e., to have the same effect on the binding. In summary, for both STAT1 and RNA Polymerase II datasets, the results of the linear least squares regression are difficult to interpret biologically. This is a typical situation where we would like to reduce the number of motifs used by the regression model. Clearly, this is not possible with the linear least squares regression approach.

#### Ridge regression

In Tables 6 and 7, we show the top ten motifs identified by the ridge regression. In the case of STAT1 (Table 6), we can see that the ridge regression and the L1-norm log linear regression identify very similar motifs. In both cases, the classical GAS motif is clearly identified as the main binding motif. Both regression methods also identify CA[TC]GTGACT[TG]C as a strengthening motif and GGAGGGCG as a weakening motif. In the case of RNA Polymerase II (Table 7), both methods are able to identify the initiator site (T[CT][TA]T[TG][AC][AT) and the downstream promoter element (A[GC][TAG]CA).

**Table 6:** List of putative STAT1 binding motifs identified by ridge regression.

*STAT1*	*Normalized coef.*
CA[TC]GTGACT[TG]C	1.
[TG]G[GTA][GC][AG]T**TT[CA]C[AGC][GA]GAA**[AC][TG]G[GA][GC]	0.89
GGAGGGCG	−0.69
[CT][TC]CA[GT]**TTCCAGGAA**[AT]T[CG][CAT]C[CT]	0.69
A[CT]**TTC[TC][TG]GGAA**	0.68
**TTC[CT][TG][GA]GAA**AT[GC][CA][CA][CAT][AT][TCG][CG][CT]	0.65
**TT[CA]C[TAG][GA]GAA**[GA]T	0.59
**TT[CA][TC][GA]GGAA**[AG]	0.58
GGACGCCG	−0.57
G[TGC][CGT][AT][TG]**TTCC[TCA][GA][GT]AA**[AG]	0.53

The classical GAS motifs are shown in boldface.

**Table 7:** List of putative RNA Polymerase II binding motifs identified by ridge regression.

*RNA Polymerase II*	*Normalized coef.*
T[AG]**A[GC][TAG]CA**[GCT]A[AC]AA	1.0
A[GA]AA[AC][CA]AA[AC]AAA	0.86
C[ACT]**[GT][CG][CT][TA]C**C[AGT]CC[TA]	0.81
C[CT][CG][AT]GGCTGG[AG]G	0.75
TTTCTGC[CT][CT]TT[GT]	0.74
GGAGGGAGGC[AG]G	0.70
AC[AC][CA][AC][AT][AG]AGAAA	0.65
T[TA]T[TC]**[CA]CAGAC**T[AT]	0.63
TTTG**T[CT][TA]T[TG][AC][AT]**T	0.62
**TT[TAC]TTT[CT]**TT[TC]TT	0.61

The known Downstream Promoter Element and Initiator site motifs are shown in boldface.

However, they differ greatly with respect to computational complexity. In [8], the authors present an algorithm for computing the L1-norm log linear regression solutions of many regularization parameters for the same computational cost as that of a single least squares fit. As a consequence, using the same STAT1 dataset, a 30-fold cross-validation takes approximately 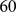 hours with the ridge regression, while it takes only 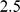 hours with the L1-norm log linear regression (i.e., 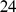 times faster). In summary, although both methods show very similar results with respect to binding motif identification, the ridge regression is slower and more difficult to use with large ChIP-Seq datasets than the L1-norm log linear regression.

#### Partial least squares regression and principal component regression

In Tables 8 and 9, we show the top ten motifs for STAT1 identified by the partial least squares regression and the principal component regression. We can see that both methods are able to identify the classical GAS motif. In Table 8, the partial least squares regression shows very similar results to the L1-norm log linear regression as both methods identify CA[TC]GTGACT[TG]C as a strengthening motif and GGAGGGCG as a weakening motif. In Table 9, the principal component regression identifies only the GAS motif and fails to identify any other motifs involved in the binding. In the case of RNA Polymerase II, both partial least squares regression (Table 10) and principal component regression (Table 11) are able to identify the initiator site and the downstream promoter element.

**Table 8:** List of putative STAT1 binding motifs identified by partial least squares regression.

*STAT1*	*Normalized coef.*
CA[TC]GTGACT[TG]C	1.0
[TG]G[GTA][GC][AG]T**TT[CA]C[AGC][GA]GAA**[AC][TG]G[GA][GC]	0.80
**TTC[CT][TG][GA]GAA**AT[GC][CA][CA][CAT][AT][TCG][CG][CT]	0.58
[CT][TC]CA[GT]**TTCCAGGAA**[AT]T[CG][CAT]C[CT]	0.56
[GA][AG]A[AG][AT][CTG][CA]A[GT][CG]T[GT][CG][CA]T[TG][CT][CGT]T	0.50
TCACA[TG]G[ACG]	0.42
GGAGGGCG	−0.41
G[TGC][CGT][AT][TG]**TTCC[TCA][GA][GT]AA**[AG]	0.41
**TT[CA]C[TAG][GA]GAA**[GA]T	0.40
A[TA]**TTCC[CT][GA]GAA**[AC]T[CG][AC]	0.39

The classical GAS motifs are shown in boldface.

**Table 9:** List of putative STAT1 binding motifs identified by principal component regression.

*STAT1*	*Normalized coef.*
[TAC]**TTCC[CA][GA][GT]AA**[AG][TA]C	1.0
T**TTCC[CT][GA]GAA**AA[CT]TC[AC]TGAA	0.94
TT**TTC[CT][AG]GGAA**[AG][GT]GG[CG][TCA][GA]GG	0.87
T**TTC[TC][TG][GA][GAT]AA**[GA]	0.86
[TC]**TTCC[AC][AG]G[AC]A**	0.85
[GA]GAACC[TC][TG]CAG**TTC[CT][AG]GGAA**	0.82
CC[CTA][CGT]T**TTC[CT]T[GA]GAA**[AG][ACT][CG]	0.82
**TTC[CT][TG][GA]GAA**AT[GC][CA][CA][CAT][AT][TCG][CG][CT]	0.81
T**TTC[CT][AGT]GGAA**A[TG][GA][GA]G[TAC][GA]G	0.80
G[CT]**TT[CA][CT][GAT][GA]GAA**[AG][TG][AGC][GA][GCA][TGA]A[CG]	0.78

The classical GAS motifs are shown in boldface.

**Table 10:** List of putative RNA Polymerase II binding motifs identified by partial least squares regression.

*RNA Polymerase II*	*Normalized coef.*
T[TG]AACAC**AGTT[TA]**	1.0
C[CT][CG][AT]GGCTGG[AG]G	0.99
G[AG]GG[CG]CCAGAGA	−0.97
[CT][CG]AG**A[GA]TCC**A[GA][CG]	−0.90
CTGG[AC]GCTG[TG][TC][ACG]	−0.89
A[AG][GA][AG]**GGA[GCA]G**A[GA]A	0.87
[CG][AT][CT]T**[GC]C[AT][CG]TCC**[AC]	0.86
GGAGGGAGGC[AG]G	0.86
A[GA]AA[AC][CA]AA[AC]AAA	0.85
[GT]GCCCAGG[CG][TG][GA]G	−0.81

The known Downstream Promoter Element and Initiator site motifs are shown in boldface.

**Table 11:** List of putative RNA Polymerase II binding motifs identified by principal component regression.

*RNA Polymerase II*	*Normalized coef.*
GCT**GG[GT][AC][CT]**[CT]ACA	−1.0
[CG]GCGGCGGCGGC	0.97
GCCCAGGCTG[CG][TA]	−0.96
CA[AC]**AG[TG][GC]C**TG[GA]G	−0.94
CTGG[TC][CT]TCAAA[GC]	−0.90
CTGG[AG]G[TG]GC[AT]G[TG]	−0.89
CTGG**A[GA]T[GT]C**A[GA][TG]	−0.87
[TC]CCA[CA]AG[CAT][AG]CTG	−0.86
**[TA]C[AC]T[GA][CG]C**CTGT[GT]	−0.84
[CA]TG[AT]CCACAGA[AT]	−0.83

The known Downstream Promoter Element and Initiator site motifs are shown in boldface.

However, the results of the partial least squares regression and the principal component regression are difficult to interpret. In the former (Table 10), different instances of the downstream promoter element have positive or negative coefficients (T[TG]AACAC**AGTT[TA]** with 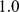, [CT][CG]AG**A[GA]TCC**A[GA][CG] with 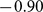, and A[AG][GA][AG]**GGA[GCA]G**A[GA]A with 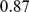). As they are instances of the same motif, we expect them to have the same sign, i.e., to have the same effect on the binding. In the latter (Table 11), all the instances of the initiator site and the downstream promoter element have negative coefficients. However, these motifs should strengthen the binding and therefore, we expect their coefficients to be positive.

The lack of interpretability of the partial least squares regression and the principal component regression lies in the fact that the regression is performed in a low-dimensional feature space. In the original motif space, the vector representation of the peak sequences has a meaning and each component of a vector measures how similar a motif is to a peak sequence. However, in the low-dimensional feature space computed by the partial least squares regression and the principal component regression, the vector components lose their biological meaning. From the computational complexity perspective, we also mention that both methods are very slow. Using the STAT1 dataset, a 30-fold cross-validation of the partial least squares regression with 10 components takes approximately 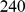 hours. In summary, the partial least squares regression and the principal component regression are able to identify the classical GAS motif for STAT1 and the initiator site and the downstream promoter element for RNA Polymerase II. However, the results are difficult to interpret biologically and do not allow identification of strengthening or weakening motifs. In addition, they are too slow to be used with large ChIP-Seq datasets.

#### Advantages of L1-norm log linear regression over other methods for TFBM identification

We considered the following regression methods for TFBM identification: L1-norm log linear regression, linear least squares regression, ridge regression, partial least squares regression, and principal component regression. In Table 12, we summarize the correlation coefficients averaged on the test sets. As we can see, all regression methods demonstrate similar performance and are able to identify the classical GAS motif for STAT1 and the initiator site and the downstream promoter element for RNA Polymerase II.

**Table 12:** Different regression methods and their correlation coefficients averaged on the test sets.

*Regression method*	*STAT1 correlation coef.*	*Pol II correlation coef.*
L1-norm log linear regression	**0.65**	**0.66**
Linear least squares regression	0.64	0.64
Ridge regression	0.64	0.64
Partial least squares regression	0.64	0.65
Principal component regression	0.63	0.52

However, they exhibit marked differences with respect to biological interpretability and computational efficiency. The results of the linear least squares regression, the partial least squares regression, and the principal component regression do not allow identification of strengthening or weakening motifs. Therefore, they are difficult to use for binding motif identification. Both L1-norm log linear regression and ridge regression solve this problem by means of regularization. However, the ridge regression is very slow compared to the L1-norm log linear regression. Therefore, the ridge regression is difficult to use with large-scale ChIP-Seq datasets. In summary, the L1-norm log linear regression is the only method that can achieve all the desired goals for our task; it identifies the transcription factor binding motifs, the regression coefficients are easy to interpret biologically, and its implementation with the LASSO algorithm is fast and efficient. This justifies our choice of the L1-norm log linear regression in PeakRegressor.

### Parameter setting

The performance of PeakRegressor depends on the choice of parameters that have to be set empirically. In this section, we explain how we choose two important parameters: the length of peak sequences and the number of motif candidates.

#### Length of peak sequences

In the dataset provided by [9], all the peaks correspond to various DNA sequences. These sequences have different lengths, ranging from 1 bp to several thousand bp. To conduct our analysis, we modify the peak sequences in the following way:

- We shorten long peak sequences for two reasons. First, when using long DNA sequences, the computations of the motif finding algorithm MEME take too much time. Second, finding good quality motifs with MEME is easier with short DNA sequences than with long ones.
- We widen short peak sequences. Due to the noisy nature of ChIP-Seq data, the motifs we are looking for may not be exactly on the provided peak sequence, but in the surrounding DNA neighborhood. Therefore, we decide to choose a uniform length for all the peak sequences. The choice of 200 bp is empirical; we try several values (100 bp, 200 bp, 400 bp, and 800 bp) and consider the one that achieves the best performance, i.e., the highest correlation coefficients (results not shown for other peak lengths).

#### Number of motif candidates

In the first step of PeakRegressor, we use MEME to find over-represented DNA motifs in the peak sequences. This step results in 800 motif candidates for STAT1 and 880 for RNA Polymerase II. Given the large number of motif candidates, we empirically observe the presence of similar motifs in the set of motif candidates. We may wonder if this redundancy could affect the prediction performance of PeakRegressor. However, we show that this is not the case.

PeakRegressor uses a regression method called L1-norm log linear regression. In contrast with other regression methods, L1-norm log linear regression achieves its best prediction performance by removing redundant or uninformative motifs from the regression model. Therefore, the removal of redundant motifs is automatically performed when using L1-norm log linear regression. Table 2 shows the set of motifs that achieve the best correlation coefficient for STAT1. We can see that some motifs are similar. For example, the motifs A[CT]**TTC[TC][TG]GGAA** , **TT[CA]C[TAG][GA]GAA**[GA]T, A[TA]**TTCC[CT][GA]GAA**[AC]T[CG][AC], and **TT[CA][TC][GA]GGAA**[AG] are short, similar motifs containing the STAT1 binding motif. In other experiments, we find that the prediction performance worsens when similar motifs are removed (results not shown). Hence, although the motifs appear similar and redundant, they actually contain complementary information for the prediction performance.

Moreover, the motif weights computed by PeakRegressor are all different (resp. 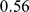, 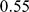, 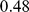, and 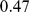). Hence, while other approaches, such as motif clustering, would consider all these motifs to be equally important, PeakRegressor is able to detect the relative importance of each motif and compute the corresponding weight. This is explained by the noisy nature of the DNA motifs found by MEME in step 1. For a given binding motif, PeakRegressor needs to use all the noisy PSSM approximations to achieve the best prediction performance. This is an important property of PeakRegressor, especially when the number of noisy motifs is very large.

### Candidate motifs and their potential rSNPs

Single or composite motifs found in the PeakRegressor system may reflect actual transcription factor binding sites. If a single nucleotide polymorphism (SNP) occurs within the sites, regulatory control of neighboring gene transcription will be perturbed, thus leading to genetic diseases in some cases [10]. Therefore, true binding sites may have SNPs less frequently than the non-binding sites. As an important verification, we check the number of known SNPs to be found within the STAT1 positions presented by PeakRegressor by using dbSNP database (http://www.ncbi.nlm.nih.gov/SNP/). We find that 0.36% (147 for 40,395 bp) of mapped positions with 10 STAT1 motifs in Table 2 on the peak sequences contains SNPs, while as much as 0.53% (17,852 for 3,344,439 bp) of all positions contains SNPs on the peak sequences. The statistical difference between the above two ratios is highly significant such as 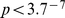 according to the hypergeometric distribution. These sites are possible candidates of rSNPs because the slight change within the motif may affect the change of gene expression level and might cause diseases.

## Materials and Methods

### PeakRegressor

**Figure 2:**
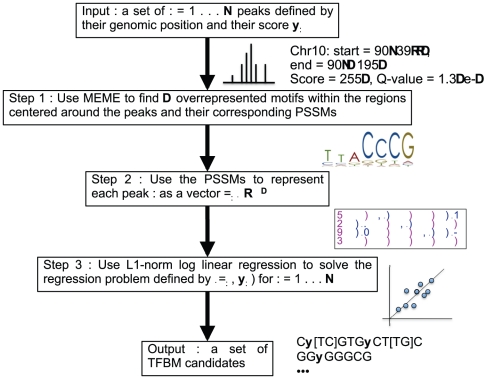
Schematic view of the workflow of PeakRegressor. PeakRegressor takes ChIP-Seq data as input and outputs a list of TFBM candidates and their weights that give the best regression accuracies.

PeakRegressor is a system to find TFBMs that are statistically important for transcription factor binding signals, by taking ChIP-Seq data as input, and outputs a list of TFBM candidates. In contrast with previous approaches, PeakRegressor uses the peak scores (provided by [9]) as a surrogate for the binding affinities. We argue that the peak scores provide more accurate approximations of the binding affinities than the methods based on transcription factor occupancy [4], [5]. Therefore, using the peak scores lead to better identification of functional TFBMs. In addition, PeakRegressor identifies not only primary TFBM candidates but also secondary motifs that may often synergistically strengthen or weaken the binding. The workflow is summarized in Figure 2.

#### Step 1

First, we define the peak sequences as the 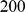-bp genomic regions centered around the peaks. Then, we sort the peak sequences according to their ascending scores. We group the peak sequences into clusters such that each cluster contains 200 peaks of consecutive scores. Then, we apply MEME (http://meme.sdsc.edu/) to each peak sequence cluster. For each sequence cluster, MEME is parameterized in ZOOPS mode to find 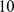 motifs of lengths 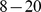.

This strategy has two advantages. First, it allows us to identify motifs that may be associated with a given binding affinity level. If a cluster contains only low (resp. high) binding affinity peaks, the corresponding sequences may contain weak (resp. strong) binding motifs, i.e., motifs that are specific to low (resp. high) binding affinity. Second, it reduces computational time by parallelizing MEME computations.

#### Step 2

In order to predict the binding affinity of the peaks, we need to represent each peak as a vector in the motif space. Let 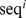 be the DNA sequence of peak 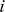. Let 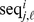 be the 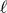-length sub-sequence of 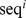, starting from position 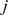. Let 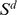 be the PSSM of motif 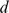. Let 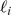 be the length of 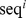 and 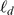 be the length of motif 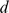. We represent peak 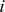 as vector 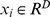, such that
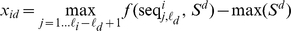
for 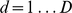. The quantity 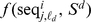 is a sum of log-odd scores, representing how well motif 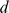 matches sub-sequence 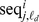. Hence, the first term of the sum, 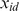, corresponds to the best match when we slide motif 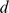 along sequence 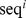. The term 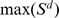 is the maximum score achievable by any sequence matching with the motif 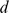. Therefore, we always have 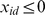, with 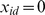 for the best possible match.

Next, we want all the 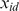 to be positive for interpretability purpose. So we simply shift their values by substracting the lowest component: 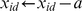, where 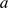 is the minimum value of the original 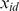. Finally, we normalize each data vector by dividing it with its euclidean norm: 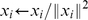.

#### Step 3

Quantities 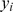 to be fitted are the log values of the peak enrichment scores, as given by PeakSeq [9]. We can now solve the regression problem defined by 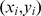 pairs for 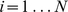. Linear regression is a simple and popular approach, but is prone to overfitting. Hence, we choose to regularize the model with L1-norm, i.e., we want to minimize the sum of squared errors and the L1-norm of the regression coefficient vector:
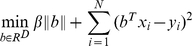
(1)where 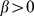 is a user-defined regularization coefficient. The L1-norm log linear regression is able to remove redundant or uninformative features, and to select a small number of features that best explain the fitted quantity [11]. In our case, the features correspond to DNA motifs and hence, the result of this step is a set of motifs that best explain the binding signal values from ChIP-Seq dataset. We use Lasso, a popular algorithm for solving L1-norm log linear regression. Lasso is available as part of the LARS package for R (http://www-stat.stanford.edu/~hastie/Papers/LARS/).

### Other regression methods

In this section, we present alternatives to the L1-norm log linear regression: linear least squares regression, ridge regression, partial least squares regression, and principal component regression. All these regression methods are used in the following way. Once a regression model is fitted to the peak dataset, we rank the regression coefficients with respect to their absolute values. Using this ranking, the top motifs are the potential TFBMs.

#### Linear least squares regression

The linear least squares regression is the simplest regression approach. It fits a linear model to the dataset by minimizing the sum of squared errors 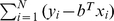. Its difference with the L1-norm log linear regression (equation 1) is the absence of a regularization term. Therefore, the linear least squares regression is more prone to overfitting when the regression problem contains more dimensions than samples.

#### Ridge regression

The ridge regression [12] minimizes 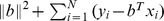, where the regularization term is 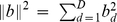, i.e., the Euclidean norm of 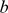. It is quite similar to the L1-norm log linear regression, and their main difference lies in the regularization term. The ridge regression seeks a solution with a low Euclidean norm. Although the Euclidean norm is a protection against overfitting, it does not favor sparse solutions (i.e., solutions with many motifs) as the L1-norm log linear regression does [11].

#### Partial least squares regression and principal component regression

The partial least squares regression [13] and the principal component regression are two approaches of the same idea; they perform linear regression using the low-dimensional data matrix 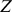 instead of the initial data matrix 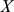. This approach avoids overfitting problems. Therefore, the partial least squares regression and the principal component regression have been widely used in problems containing several dimensions (i.e., motifs) and few samples (i.e., peaks).

In the principal component regression, the low-dimensional data matrix 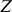 contains the most information about the initial data matrix 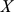 (according to the singular value decomposition of 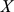). In the partial least squares regression, the low-dimensional data matrix 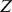 is calculated using both the initial data matrix 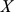 and the peak score vector 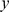. In both cases, linear regression is performed using 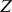 instead of the initial data matrix 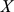. Both partial least squares regression and principal component regression are available as part of the PLS package for R (http://mevik.net/work/software/pls.html). Once the regression coefficients have been computed in the low-dimensional space, they are mapped back in the original motif space. Then, these coefficients can be used to identify potential binding motifs.

### Input ChIP-Seq datasets

The ChIP-Seq dataset we used is provided by [9] and is publicly available (http://www.camda2009.org/). The dataset provides various information about each peak, including the peak score, the peak center (for STAT1), and the Q-value that reflects the significance of the peak. The Q-values are derived from the P-values. First, they compute the P-values that reflect the significance of peak enrichment in the number of DNA tags, compared to control samples. These P-values are computed using the binomial distribution. Then, to account for multiple hypothesis testing, the Q-values are derived from the P-values. See [9] for more details.

For STAT1, we use 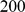-bp windows around the peak centers to define the peak sequences. For RNA Polymerase II, the peak centers are not available and thus, we use the peak start and peak end coordinates to define the peaks. When the length of the resulting sequence is less than 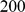 bp, we enlarge it in both directions in order to reach 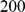 bp length. When the length is more than 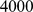 bp, we trim it in both directions in order to reach 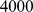 bp length. As a result, all the RNA Polymerase II peak sequence lengths lie between 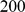 and 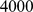 bp.

### Evaluation of prediction performance

PeakRegressor predicts the peak scores and therefore, we have two different values for each peak. The “true” peak score is the score provided by [9], and is derived from the frequency of reads of ChIP-Seq data. The predicted score is computed by PeakRegressor using the peak sequence information. Ideally, the predicted score should be equal to the true score. We use correlation coefficients to evaluate the prediction quality of PeakRegressor.

### Experimental protocol

For L1-norm log linear regression and ridge regression, we have to set the regularization parameter 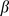. First, we define 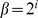 for 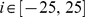. Then for each value of 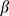, we perform a 30-fold cross-validation. In each fold, we split the dataset into a training set and a test set, with a 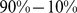 ratio. The optimal value for 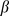 is the one which corresponds to the lowest prediction error on the test set. All the results of L1-norm log linear regression and ridge regression are averaged over the 30-fold cross-validation.

For partial least squares regression and principal component regression, the experiments were limited by the slowness of both methods. First we have to set the number of components 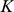 used for regression. We tried 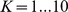, and performed a 30-fold cross-validation for each value of 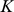. In each fold, we split the dataset into 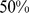 for training and 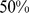 for testing. All the results of partial least squares regression and principal component regression are averaged over the 30-fold cross-validation.
